# Unveiling Proteomic Signatures of Tau Pathogenesis through Label‐Free Mass Spectrometry Analysis

**DOI:** 10.1002/alz.092740

**Published:** 2025-01-03

**Authors:** Xuemei Zeng, Yijun Chen, Anuradha Sehrawat, Eric E Abrahamson, Julia Kofler, William R Paljug, Milos D Ikonomovic, Thomas K Karikari

**Affiliations:** ^1^ University of Pittsburgh, Pittsburgh, PA USA; ^2^ VA Pittsburgh Healthcare System, Pittsburgh, PA USA; ^3^ University of Pittsburgh School of Medicine, Pittsburgh, PA USA

## Abstract

**Background:**

Neurofibrillary tangles (NFT), consisting of hyperphosphorylated tau aggregates, are one of the major pathological hallmarks of Alzheimer’s disease (AD). The burden of NFTs correlates with cognitive decline, and in vivo detection of NFT may help predict the clinical progression of AD. Mass spectrometry‐based proteomic analysis of brain regions affected by NFTs holds the potential to unveil the molecular mechanisms underlying tau pathogenesis and uncover novel diagnostic/prognostic biomarkers and therapeutic targets.

**Method:**

Post‐mortem frozen samples of inferior temporal and middle frontal cortex were collected from 9 cases with different severity of tau pathology assessed by Braak NFT stage. Proteins were fractionated into three fractions using sequential extraction with lysis buffers with different solubility strengths (TBS, Na2CO3, and Urea), trypsin‐digested, and analyzed using label‐free nano‐flow liquid chromatography‐tandem mass spectrometry (nano LC‐MS/MS). Linear mixed models were employed to assess the significance of proteins showing a differential abundance in tissues with Braak NFT stages 0‐III and IV‐VI or displaying Braak stage‐dependent solubility changes.

**Result:**

A total of 64908 peptides originating from 5475 protein groups were identified through the analysis of 18 brain tissue samples. Differential proteomic analysis revealed several proteins with significantly varied abundance in tissues with different severity of tau pathology. The most significant proteins among these include COL25A1, SNRNP70, MDK, ASPRV1, CLMN, DOCK5, HTRA1, LNPEP, SQSTM1 and Aβ peptides. Additionally, a number of proteins exhibited Braak‐stage dependent solubility change; a significant subset of these proteins, including SNRNP70, SNRPGP15, PRPF6, SNRPE, SNRPD1, SNRPB, and SNRPD3, is involved in the spliceosome‐mediated mRNA splicing.

**Conclusion:**

Our study reveals numerous proteins exhibiting a significant association with tau pathology. These results align well with published findings that suggest the involvement of alternative splicing in reprogramming gene expression in the pathogenesis of AD. Further validation will be needed to assess their potential as therapeutic targets and/or diagnostic/prognostic biomarkers for AD pathologies.

